# Comparisons of home-based arts engagement across three national lockdowns during the COVID-19 pandemic in England

**DOI:** 10.1371/journal.pone.0273829

**Published:** 2022-08-31

**Authors:** Hei Wan Mak, Feifei Bu, Daisy Fancourt

**Affiliations:** Department of Behavioral Science and Health, Institute of Epidemiology & Health Care, University College London, United Kingdom; Polytechnic Institute of Coimbra: Instituto Politecnico de Coimbra, PORTUGAL

## Abstract

Between March 2020 and March 2021, the United Kingdom (UK) experienced three lockdowns due to the COVID-19 pandemic. Given the evident association between arts engagement and wellbeing, this study was designed to compare the predictors and patterns of home-based arts engagement during these lockdowns. Data analysed in this study were from the UK COVID-19 Social Study run by University College London. Multinomial logistic regression was used to identify predictors of arts engagement and compare (i) respondents’ engagement levels during the first lockdown in April/May 2020 and their levels in pre-pandemic times (N = 23,086), (ii) their engagement levels during the second lockdown in November/December 2020 with their levels during the first lockdown (N = 11,481), and (iii) their engagement levels during the third lockdown in January/February 2021 with their levels during the first lockdown (N = 13,270). During first lockdown, 1 in 4 increased their arts engagement and 1 in 6 decreased it. Of those who increased, 2 in 5 maintained or further increased their engagement in subsequent lockdowns, but just 7% of those who had decreased their engagement increased it. Younger adults (aged 18–29) showed initial increases in first lockdown, whilst people who were not employed and those with a physical health condition showed decreases and people with a mental health condition showed changes during the first lockdown (both increases and decreases). Females and people with higher education showed continuous changes across the three lockdowns, with women being more likely to increase their engagement and those with higher education being less likely to decrease. People of ethnic minorities and those with higher income declined their engagement in the third lockdown. This study provides insight into levels of arts engagement across the three national lockdowns in the UK and suggests that the pandemic may have affected long-term cultural behaviours for some groups.

## Introduction

Between March 2020 and March 2021, the United Kingdom (UK) experienced three lockdowns due to the COVID-19 pandemic. Many individuals across the country spent a significant amount of time in their home. For this reason, the way in which people spent their time indoors became critical to their wellbeing. Recent research has shown that arts and cultural engagement might have played a role in supporting mental health during these lockdowns. Arts and cultural engagement involve a range of activities that usually comprise novelty, creativity or imaginative experiences, require specialised skills in its production, or provoke emotional response. Historically, such activities have been broadly grouped into categories such as performing arts (e.g. singing, dancing, acting), visual arts and crafts (e.g. painting, sewing, photography), literature (e.g. writing, reading), and cultural engagement (e.g. going to museums, galleries, arts exhibitions, the theatre). However, it is increasingly recognised that arts and cultural activities in fact comprise dozens of ‘active ingredients’ relating to the artistic content itself as well as the people involved and the context in which it is engaged in and received [1]. As such, some activities may share multiple common ingredients whilst others (even within similar ‘categories’ e.g. visual arts) may be experientially quite different.

In the UK, a study of 55,000 adults showed that increased time spent on hobbies such as painting, creative writing, woodwork and other creative activities was associated with greater levels of life satisfaction and decreases in depressive and anxiety symptoms during the pandemic [2]. Individuals who spent more time listening to music (vs less time) were found to have higher levels of life satisfaction, whereas those who spent more time on watching TV/movies/streaming videos video games (vs less time) had lower life satisfaction. Young adults whose routines had been heavily affected by the pandemic (due to school closures and a sudden transition to online education) were more likely to report higher efficacy of music in obtaining feelings of enjoyment, maintaining good mood and distracting themselves from the pandemic [3]. Further, for individuals experiencing extreme stress during the pandemic, such as frontline health and social care professionals, arts activities were shown to help cope with the uncertainty and challenges of the pandemic [4], and to provide mental health support [5]. Specifically, research suggests that the role of the arts in supporting coping and emotion regulation was key to these psychological benefits. For instance, a study examining over 19,000 adults in the UK showed that some people used the arts to help avoid negative emotions, approach problems or for self-development during the first lockdown [6]. This builds on research prior to the pandemic suggesting positive mental health and wellbeing benefits [7], but highlights how such benefits can transfer to pandemic circumstances. This provides evidence for arts engagement as an effective way of mitigating worsening mental health outcomes during the COVID-19 pandemic [8].

Several studies have suggested that many people increased their engagement in home-based arts activities during the first lockdown in 2020 [6,9,10]. For instance, a study of 19,000 individuals in the UK showed that around 22% took part in more arts activities at home during the first lockdown [6]. Similarly in Spain, a survey of over 1,500 respondents showed that nearly half of them spent more time listening to music, over a quarter spent more time on singing and a third of them spent more time on dancing and playing an instrument soon after the lockdown compared to before the pandemic [10]. However, as the first lockdown was eased, evidence from the UK suggested that arts engagement began to decline. A study of over 29,000 adults in the UK identified 5 unique classes of growth trajectories during and following the first lockdown, with patterns of engagement aligning with changes in social restrictions [11]. Two of these classes indicated stable levels of either high engagement or very low engagement during and following the first lockdown, whereas two other classes showed initial increases in arts engagement during the first lockdown followed by declines as restrictions were eased. For these classes, the resumption of usual activities such as work, socialising, increased leisure opportunities and returning to school and workplaces might be responsible for the decline. The remaining class showed the opposite pattern of declines followed by an increase as restrictions were lifted, with women and people with higher education levels most likely to be in this group perhaps due to greater health awareness of the arts [11]. However, most of these studies were conducted during and following the first lockdown. What remains unclear is how arts engagement patterns changed over subsequent lockdowns across the latter half of 2020 and into 2021. For example, it is currently unknown whether home-based arts engagement increased again as people were again confined to their homes for subsequent lockdowns, or whether the looser lockdown restrictions (for example permitting children to go to school, more social contact outdoors, and more adults to work away from the home) meant that other activities displaced leisure time available for arts engagement. Notably, mental health and wellbeing did worsen again as virus levels rose, both in the lead up to and during lockdowns in the autumn of 2020 and start of 2021. Therefore, it is important to ascertain whether arts engagement was still utilised as an activity to support coping during these periods.

Further, it is crucial to identify whether patterns of engagement were different amongst different demographic groups. There is a well-reported social gradient in arts engagement outside of pandemic circumstances [12–14]. In general, young people, females, people of white ethnic, those with higher education levels, and people with good health are more likely to participate in arts activities. However, research suggests that audiences for home-based arts engagement were expanded during the first lockdown. While some of the usual “engagers” continued to engage (particularly female and those with higher education levels), other groups such as people with lower household income and people with mental health conditions (who usually have lower engagement levels) were found to engage more in arts activities during the first lockdown [6,11]. This suggests that new profiles of arts audiences might have emerged during the pandemic. But it is unknown whether this broadening of audiences continued across later lockdowns. Understanding the predictors of arts engagement across different phases of the pandemic is important to identify whether the pandemic has led to long-term shifts in audience profiles, or whether barriers to engagement amongst certain demographic groups remain. This will provide important implications for the arts and cultural sector in the design and delivery of their work (including the possibility of partly transitioning the arts to online platforms to reach our wider audiences), as well as filling in the literature gap in understanding how arts engagement across different demographics could be altered when social contexts were changed.

In this light, the present study was designed to explore the predictors of home-based arts engagement across three national lockdowns during the COVID-19 pandemic in the UK. Specifically, we sought to understand how levels of arts engagement across socio-demographic and health profiles changed during (1) the first lockdown in April/May 2020 compared to pre-pandemic periods, (2) the second lockdown in November/December 2020 compared to the first lockdown, and (3) the third lockdown in January/February 2021 compared to the first lockdown. Due to varying COVID-19 pandemic restrictions in different nations across the UK, we only considered respondents who resided in England.

## Materials and methods

Data were drawn from the COVID-19 Social Study; a large ongoing panel study of the psychological and social experiences of over 70,000 adults (aged 18+) in the UK during the COVID-19 pandemic. The study commenced on 21st March 2020 and involved online weekly data collection until August 2020, followed by monthly data collection from participants for the duration of the COVID-19 pandemic in the UK. The study did not use a random sample design, but it does contain a heterogeneous sample. Participants were recruited using three primary approaches. First, convenience sampling was used, including promoting the study through existing networks and mailing lists (including large databases of adults who had previously consented to be involved in health research across the UK), print and digital media coverage, and social media. Second, more targeted recruitment was undertaken focusing on (i) individuals from a low-income background, (ii) individuals with no or few educational qualifications, and (iii) individuals who were unemployed. Third, the study was promoted via partnerships with third sector organisations to vulnerable groups, including adults with pre-existing mental health conditions, older adults, carers, and people experiencing domestic violence or abuse. The study was approved by the UCL Research Ethics Committee [12467/005] and all participants gave written informed consent. The study protocol and user guide (which include full details on recruitment, retention, data cleaning and sample demographics) are available at https://osf.io/jm8ra/s.

In this study, we considered three samples across three national lockdowns in the UK. The first lockdown commenced on 23^rd^ March 2020 with people being ordered to “stay at home”. The restrictions began to ease in England on 10^th^ May 2020 with more movements around the country permitted (including unlimited outdoor exercise and return to work). The first sample was the respondents who completed a one-off module between 21^st^ and 28^th^ May 2020 which included a question on their arts engagement during April/May (N = 25,742). On 5^th^ November 2020, the government announced a second national lockdown. We therefore considered respondents who had completed both the one-off May module and the survey collected between 12^th^ November and 3^rd^ December 2020 (the end of the second lockdown) (N = 17,107). Finally, on 6^th^ January 2021, England entered its third national lockdown. The third sample was the respondents who completed both the one-off May module and the survey collected between 13^th^ January 2021 and 24^th^ February 2021 (2 weeks before the third lockdown measures began to ease in March) (N = 19,353). For all three samples, we only considered respondents who provided responses to all measures. This provided us three final analytic sample sizes of N = 23,086, N = 11,481, and N = 13,270 participants in three different lockdowns respectively.

### Measures

Three measures of arts engagement levels across three national lockdowns were considered in this study. Arts engagement was defined as creative activities that respondents had engaged in the past week. These included singing, playing a musical instrument, painting, drawing, printmaking or sculpture, reading books, stories or poetry, textile crafts (e.g. embroidery, crocheting or knitting), wood crafts (e.g. carving or furniture making), other crafts (e.g. pottery, calligraphy or jewellery making), creative writing, dancing, photography, creative digital artworks or animations, making films or videos, listening to music, and “other creative activity”. Participants were asked to consider all of these activities together and report how their levels of engagement changed during the first lockdown (April/May 2020) compared to prior to the pandemic. Reponses ranged from “less than usual”, “about the same”, to “more than usual”. In the subsequent monthly follow-ups, the question about levels of arts engagement was repeated, and respondents were asked to compare their current levels of arts engagement to the levels during the first lockdown in April/May 2020. This time, responses ranged from “less than during lockdown in April/May 2020”, “about the same”, “more than during lockdown in April/May 2020”, to “not applicable/never do the activity”. To be more accurate with the measures, respondents who reported not engaging in any of the arts activities in the first lockdown and also reported “not applicable/never do the activity” in the subsequent lockdowns were included in the “about the same” category for second or third lockdown. Alternatively, for respondents who reported engaging in any of the arts activities in the first lockdown but reported “not applicable/never do the activity” in the second or third lockdown, they were included in the “less than during lockdown in April/May 2020” category.

A range of demographic, socio-economic and health factors were considered as potential predictors of levels of arts engagement. All were measured at baseline. These included age groups (18–29, 30–59, 60+), gender (female vs male), ethnicity (white ethnic vs ethnic minorities), whether or not living with children, partnership status (married/in a relationship vs not married/not in a relationship), living area (city, large/small town, village/hamlet/isolated dwelling), employment status (employed vs unemployed/economically inactive e.g. student, retired, homemakers, etc.), education levels (up to GCSE/CSE/O-levels or equivalent, post-16 vocational or A-levels qualifications or equivalent, degree or above), annual household income (<£30,000 vs ≥£30,000), and living environment (living in an overcrowded household vs not living in an overcrowded household). We also considered two health-related factors–diagnosed mental health condition (clinically diagnosed anxiety, depression or other psychiatry condition; yes vs no) and diagnosed physical health condition (high blood pressure, diabetes, heart disease, lung disease e.g. asthma or COPD, cancer, other clinically diagnosed chronic physical health condition or disability; yes vs no).

### Analysis

To understand the changes in arts engagement across three national lockdowns, we used multinomial logistic regression to calculate the relative risk ratio (RRR) and 95% confidence intervals (CIs) to predict how likely participants were to have changed their levels of arts engagement. Participants who reported their engagement levels were about the same as usual or as during lockdown in April/May were considered as the reference comparison group. A RRR that is greater than one indicates an increased risk of being in any other groups (i.e. more than usual or during lockdown in April/May, or less than usual or during lockdown in April/May), while a RRR that is lower than one suggests a decreased risk of being in these groups.

To balance the data against population demographics, we weighted data to match the core demographic features of the target population (namely gender, age groups, ethnicity and education) obtained from the Office for National Statistics (ONS; 2020) [15]. This was done separately for three samples by using the Stata user-written command ‘ebalance’ [16].

## Results

The weighted demographic, socio-economic and health profiles of the three samples were similar (Table 1). On average, more than half of our samples were aged 30–59 (51–53%), 50–51% were female, and around 13–14% were from an ethnic minority background. Two-third of our samples were married or in a relationship, nearly 60% were employed, and 37% had a degree or above education qualification. About one in five had a diagnosed mental health condition and two in five had a physical health condition.

**Table 1:** Descriptive statistics (weighted).

	1^st^ lockdown (Apr/May 2020) N = 23,086	2^nd^ lockdown (Nov/Dec 2020) N = 11,481	3^rd^ lockdown (Jan/Feb 2021) N = 13,270
**Demographic backgrounds**			
Ages 18–29	18.4%	17.4%	17.1%
Ages 30–59	52.1%	51.3%	52.5%
Ages 60+	29.5%	31.4%	30.5%
Female	51.1%	50.4%	50.5%
Male	48.9%	49.6%	49.5%
White ethnic	86.0%	86.4%	87.3%
Ethnic minorities	14.0%	13.6%	12.7%
Living with children	24.1%	20.6%	21.1%
Not living with children	75.9%	79.4%	78.9%
Married/in a relationship	66.2%	65.7%	66.8%
Not married/not in a relationship	33.9%	34.3%	33.2%
Living in city	35.9%	34.7%	34.2%
Living in large or small town	45.3%	45.8%	46.3%
Living in village/hamlet/isolated dwelling	18.8%	19.6%	19.5%
**Socio-economic position**			
Employed	59.3%	58.1%	58.5%
Unemployed/economically inactive	40.7%	41.9%	41.5%
Up to GCSE/CSE/O-levels or equivalent	30.6%	31.3%	31.5%
Post-16 vocational or A-levels qualifications or equivalent	32.5%	31.5%	31.6%
Degree or above	36.8%	37.3%	36.9%
Household income <£30,000	44.1%	44.9%	45.1%
Household income ≥£30,000	55.9%	55.1%	54.9%
Living in overcrowded households	15.3%	13.3%	13.8%
Not living in overcrowded households	84.7%	86.7%	86.2%
**Health condition**			
Diagnosed mental health condition	19.5%	17.4%	17.8%
No diagnosed mental health condition	80.5%	82.6%	82.3%
Diagnosed physical health condition or disability	40.1%	41.6%	40.7%
No diagnosed physical health condition or disability	59.9%	58.4%	59.3%

**Fig 1:**
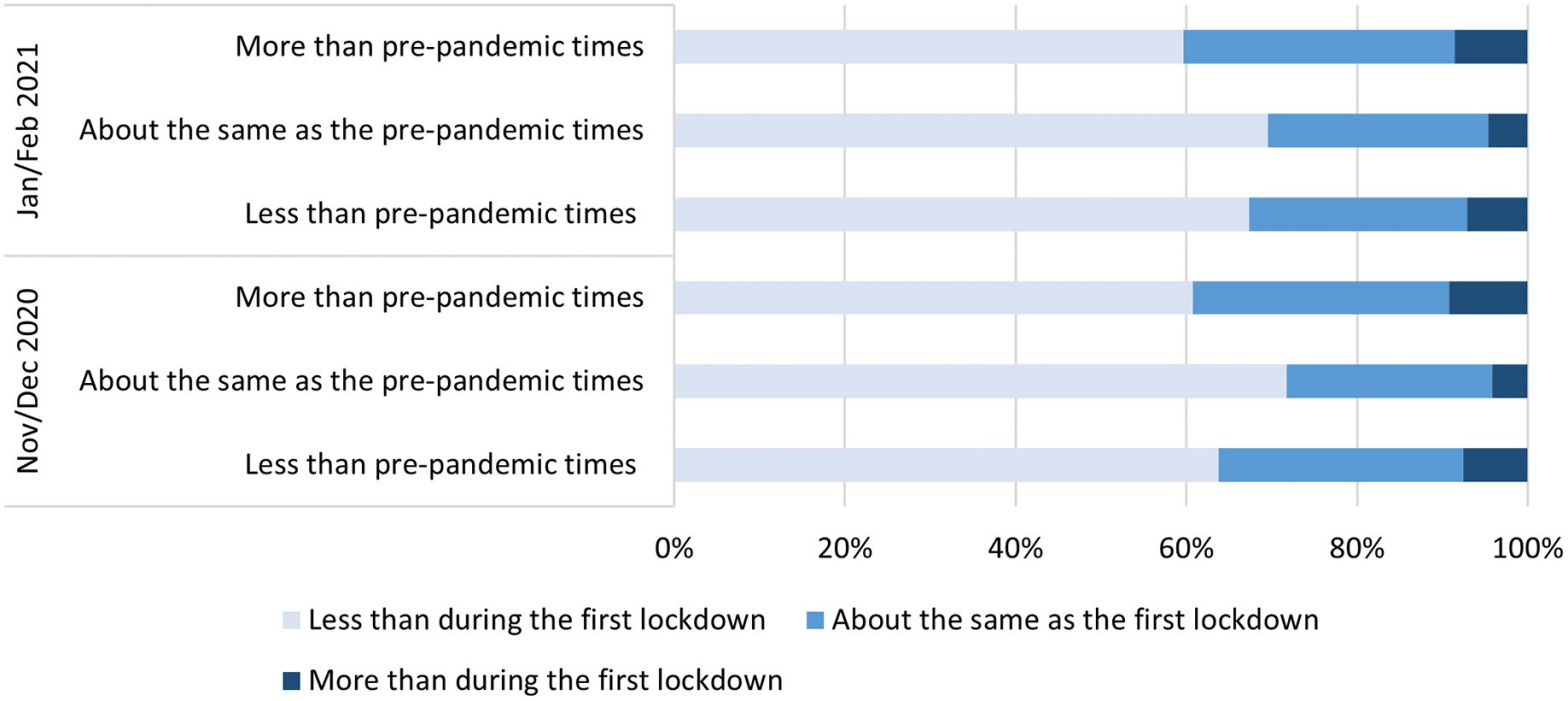
Changes in arts engagement levels between first lockdown in April/May 2020, second lockdown in November/December 2020, and third lockdown in January/February 2021.

During the first lockdown in April/May 2020, about 24% reported engaging in arts activities more than before the pandemic, whilst 16% engaged less and 59% engaged about the same. Of respondents who reported engaging more in arts activities during the first lockdown than before the pandemic, 39% had either retained high levels of engagement or further increased their engagement during the second lockdown in November/December 2020 (with 9% specifically reporting a further increase). Of those who engaged less during the first lockdown than before the pandemic, 93% had either retained low levels of engagement or reduced their engagement during the second lockdown (with 64% reporting a decrease) (Fig 1).

Similar results were found during the third lockdown in January/February 2021 as during the second lockdown. Amongst respondents who engaged more arts activities during the first lockdown than prior to the pandemic, 40% reported either having about the same engagement levels or having increased their engagement during the third lockdown (with 9% reporting an increase). Of those who reduced their engagement levels between before the pandemic and during the first lockdown, 93% reported either retaining low levels of engagement or further reducing their engagement during the third lockdown (with 67% reporting a decrease) (Fig 1).

### Demographic backgrounds

When comparing the levels of arts engagement during the first lockdown with the levels during usual times (i.e. prior to the pandemic), young adults aged 18–29 had 54% higher RRR of engaging more with the arts than adults aged 30–59, whereas older adults aged 60+ had 21% lower RRR of engaging more relative to staying the same. However, no age differences in changes of arts engagement were found in the second and third lockdowns (Tables 2–4). Descriptive statistics for all three age groups show that the majority of those who had decreased their engagement during first lockdown continued to decrease it in the third lockdown, in line with the patterns shown for the entire sample (S1 File–contains all the supporting figures).

**Table 2:** Multinomial logistic regressions comparing arts engagements in the first lockdown (April/May 2020) with engagements in pre-pandemic periods (N = 23,086).

	Less than usual vs about the same			More than usual vs about the same		
	RRR	95% CI	P-value	RRR	95% CI	P-value
**Demographic backgrounds**						
Ages 18–29	1.02	0.77–1.35	0.893	**1.54**	**1.27–1.88**	**0.000**
Ages 60+ (ref: ages 30–59)	1.16	0.97–1.39	0.115	**0.79**	**0.68–0.93**	**0.005**
Female (ref: male)	**1.63**	**1.39–1.90**	**0.000**	**2.15**	**1.89–2.46**	**0.000**
White ethnic (ref: ethnic minorities)	0.90	0.65–1.25	0.526	1.02	0.81–1.30	0.859
Living with children (ref: not living with children)	1.00	0.83–1.20	0.975	1.09	0.93–1.27	0.289
Married/in a relationship (ref: not married/not in a relationship)	0.94	0.80–1.11	0.485	1.09	0.95–1.25	0.215
Living in large or small town	**0.83**	**0.70–0.98**	**0.027**	0.95	0.83–1.10	0.505
Living in village/hamlet/isolated dwelling	0.83	0.67–1.01	0.068	0.95	0.81–1.12	0.554
**Socio-economic position**						
Employed (ref: unemployed/economically inactive)	**0.69**	**0.57–0.83**	**0.000**	1.13	0.97–1.32	0.113
Post-16 vocational or A-levels qualifications or equivalent	1.18	0.96–1.45	0.108	1.14	0.95–1.36	0.156
Degree or above (ref: up to GCSE/CSE/O-levels or equivalent)	**1.47**	**1.20–1.81**	**0.000**	**1.46**	**1.25–1.72**	**0.000**
Household income ≥£30,000 (ref: household income <£30,000)	1.18	0.99–1.41	0.072	1.07	0.92–1.23	0.393
Not living in overcrowded households (ref: living in overcrowded households)	0.97	0.74–1.27	0.824	0.98	0.80–1.20	0.844
**Health condition**						
Diagnosed mental health condition (ref: no condition)	**1.74**	**1.45–2.09**	**0.000**	**1.31**	**1.11–1.55**	**0.002**
Diagnosed physical health condition or disability (ref: no condition or disability)	**1.26**	**1.07–1.49**	**0.006**	0.97	0.86–1.10	0.684
**Constant**	**0.19**	**0.12–0.29**	**0.000**	**0.17**	**0.12–0.25**	**0.000**

Note: Bold values denote statistical significance at the p < *0*.*05* level.

**Table 3:** Multinomial logistic regressions comparing arts engagements in the second lockdown (November/December 2020) with engagements in the first lockdown in April/May (2020) (N = 11,481).

	Less than during lockdown in Apr/May vs about the same			More than during lockdown in Apr/May vs about the same		
	RRR	95% CI	P-value	RRR	95% CI	P-value
**Demographic backgrounds**						
Ages 18–29	1.08	0.67–1.77	0.743	1.52	0.89–2.61	0.125
Ages 60+ (ref: ages 30–59)	0.83	0.67–1.04	0.104	0.85	0.59–1.22	0.376
Female (ref: male)	**0.71**	**0.57–0.88**	**0.002**	**2.76**	**1.84–4.14**	**0.000**
White ethnic (ref: ethnic minorities)	0.75	0.45–1.24	0.261	0.88	0.43–1.78	0.720
Living with children (ref: not living with children)	**0.75**	**0.58–0.96**	**0.022**	**0.56**	**0.37–0.83**	**0.004**
Married/in a relationship (ref: not married/not in a relationship)	0.89	0.71–1.13	0.339	1.15	0.79–1.66	0.466
Living in large or small town	1.23	0.99–1.53	0.061	1.09	0.78–1.51	0.623
Living in village/hamlet/isolated dwelling (ref: living in city)	1.01	0.79–1.28	0.941	0.89	0.61–1.30	0.537
**Socio-economic position**						
Employed (ref: unemployed/economically inactive)	1.13	0.87–1.48	0.359	0.95	0.63–1.44	0.821
Post-16 vocational or A-levels qualifications or equivalent	**0.64**	**0.49–0.83**	**0.001**	0.99	0.62–1.58	0.967
Degree or above (ref: up to GCSE/CSE/O-levels or equivalent)	**0.48**	**0.38–0.61**	**0.000**	0.98	0.64–1.51	0.923
Household income ≥£30,000 (ref: household income <£30,000)	1.06	0.81–1.40	0.666	1.01	0.68–1.49	0.970
Not living in overcrowded households (ref: living in overcrowded households)	0.97	0.65–1.44	0.874	1.37	0.76–2.47	0.296
**Health condition**						
Diagnosed mental health condition (ref: no condition)	1.27	0.99–1.63	0.060	1.47	0.99–2.18	0.057
Diagnosed physical health condition or disability (ref: no condition or disability)	0.82	0.65–1.05	0.111	1.00	0.73–1.39	0.980
**Constant**	**6.42**	**3.48–11.87**	**0.000**	**0.09**	**0.04–0.22**	**0.000**

Note: Bold values denote statistical significance at the p < *0*.*05* level.

**Table 4:** Multinomial logistic regressions comparing arts engagements in the third lockdown (January/February 2021) with engagements in the first lockdown in April/May (2020) (N = 13,270).

	Less than during lockdown in Apr/May vs about the same			More than during lockdown in Apr/May vs about the same		
	RRR	95% CI	P-value	RRR	95% CI	P-value
**Demographic backgrounds**						
Ages 18–29	0.94	0.63–1.40	0.756	1.12	0.67–1.86	0.673
Ages 60+ (ref: ages 30–59)	0.94	0.78–1.14	0.548	1.07	0.76–1.51	0.682
Female (ref: male)	**0.69**	**0.57–0.83**	**0.000**	**2.49**	**1.75–3.56**	**0.000**
White ethnic (ref: ethnic minorities)	**0.68**	**0.49–0.95**	**0.025**	0.70	0.36–1.34	0.279
Living with children (ref: not living with children)	0.84	0.66–1.05	0.131	0.83	0.57–1.19	0.304
Married/in a relationship (ref: not married/not in a relationship)	0.89	0.74–1.07	0.215	0.84	0.61–1.15	0.267
Living in large or small town	**1.26**	**1.04–1.53**	**0.020**	0.94	0.67–1.31	0.720
Living in village/hamlet/isolated dwelling (ref: living in city)	0.98	0.78–1.23	0.879	**0.68**	**0.48–0.98**	**0.037**
**Socio-economic position**						
Employed (ref: unemployed/economically inactive)	1.08	0.87–1.33	0.492	0.95	0.63–1.44	0.811
Post-16 vocational or A-levels qualifications or equivalent	0.78	0.60–1.00	0.052	1.28	0.82–2.00	0.286
Degree or above (ref: up to GCSE/CSE/O-levels or equivalent)	**0.56**	**0.45–0.70**	**0.000**	1.01	0.68–1.52	0.946
Household income ≥£30,000 (ref: household income <£30,000)	**1.28**	**1.06–1.55**	**0.012**	1.34	0.95–1.90	0.100
Not living in overcrowded households (ref: living in overcrowded households)	0.97	0.71–1.33	0.843	0.83	0.48–1.45	0.520
**Health condition**						
Diagnosed mental health condition (ref: no condition)	1.15	0.90–1.47	0.255	1.43	0.99–2.06	0.058
Diagnosed physical health condition or disability (ref: no condition or disability)	0.96	0.80–1.16	0.690	0.92	0.68–1.25	0.589
**Constant**	**5.04**	**3.06–8.28**	**0.000**	**0.18**	**0.07–0.46**	**0.000**

Note: Bold values denote statistical significance at the p < *0*.*05* level.

Females were more likely to experience changes in arts engagement (either increasing or decreasing) during the first lockdown. However, they were more likely to increase their arts engagement across the subsequent lockdowns, with around 2 to 3 times the risk of increasing their engagement levels compared to their male counterparts (Tables 2–4; S1 File). This was in opposition to ethnicity. Whilst no ethnic differences were found when comparing the first lockdown with the usual times, as well as when comparing the second lockdown with the first lockdown, individuals of white group were 32% less likely to have reduced their engagement levels in the third lockdown (Tables 2–4; S1 File).

There was little evidence of changes in patterns of arts engagement either from before the pandemic to during first lockdown, or when comparing the first and subsequent lockdowns relating to whether or not people were living with children (those with children were in fact less likely to make any changes during the second lockdown) or living areas (with only some small transient changes reported). No associations were found between partnership status and changes in engagement in any of the lockdowns (Tables 2–4).

### Socio-economic position

When comparing to the pre-pandemic times, employed individuals were less likely to reduce their engagement in the arts than the not employed during the first lockdown (RRR = 0.69). However, this pattern of change was not seen in the second or third lockdown when comparing with the first lockdown (Tables 2–4; S1 File). Individuals with a degree or above qualification were more likely to report a change in their engagement levels during the first lockdown, both increasing (RRR = 1.46) and decreasing (RRR = 1.47) engagement. Higher educational qualifications were also associated with a lower risk of decreasing arts engagement in later lockdowns relative to the first lockdown (second vs first lockdown: RRR = 0.48; third vs first lockdown: RRR = 0.56) (Tables 2–4; S1 File).

Higher (vs lower) household income was not associated with changes in arts engagement during first or second lockdowns, but people with a higher household income had 28% higher RRR of engaging less with the arts during the third lockdown relative to the first lockdown (S1 File). No associations were found between living in overcrowded accommodation and levels of engagement in any of the lockdowns (Tables 2–4).

### Health conditions

Finally, people with a diagnosed mental health condition were more likely to have indicated a change in their levels of engagement during the first lockdown, both increasing (RRR = 1.31) and decreasing (RRR = 1.74). People with a physical health condition had a 26% higher relative risk of reducing their arts engagement in the first lockdown than those without a condition. No mental or physical health differences in changes of arts engagement were found in the second or third lockdown (Tables 2–4; S1 File).

## Discussion

This study was one of the first to explore changes in arts engagement across three national lockdowns in England. Overall, of respondents who engaged more with arts activities during the first lockdown in April/May 2020 than before the COVID-19 pandemic, around two-fifths continued to maintain or even further increased this engagement during the second lockdown in November/December 2020 and the third lockdown in January/February 2021. Conversely, for those who engaged less during the first lockdown, just 7% increased their engagement in a subsequent lockdown. These changes in arts engagement were found to be related to people’s demographic, socio-economic and health profiles. Specifically, we found that younger adults (aged 18–29) were more likely to show initial increases in first lockdown, whilst people who were not in employment and those with a physical health condition were more likely to show decreases during first lockdown and people with a mental health condition showed changes during the first lockdown (both increases and decreases). Other groups showed continuous changes across the three lockdowns, with women showing patterns of increase across lockdowns and people with higher educational qualifications being less likely to decrease their engagement in later lockdowns. There were also groups who did not show any initial subgroup differences in early lockdowns, but did in the third lockdown, including people of ethnic minorities and those with higher income being more likely to decrease their engagement. A range of other factors were not shown to be related to changes in arts engagement over the three lockdowns such as partnership status and household overcrowding.

Notably, one of the most consistent predictors of changes in arts engagement across the three lockdowns was gender: the relative risk of increasing engagement in the arts during the second and third lockdowns for females were nearly three-fold than for males, suggesting that the gender difference in arts engagement was enlarged during the pandemic. This is in line with previous studies, both before and during the pandemic showing that females were more likely than males to engage in the arts [6,11–14], but it extends these findings by showing that increases during the first lockdown were followed by continued or further increases in second and third lockdowns. Explanations relating to gender expectations, opportunities and coping strategies can help explain the consistently higher engagement rates amongst females. For instance, gender-roles socialisation and gender identity theories in Sociology suggest that the feminine connotations of the arts have encouraged the cultivation of arts engagement amongst females [17]. It has also been suggested that females may be more likely to invest in cultural capital by participating in arts and cultural activities in order to compensate for their less favourable social position [18]. Finally, a study focusing on the arts engagement during the first lockdown in the UK shows that females tended to use the arts to avoid and detach from negative emotions [6]. This suggests that the persistence of the increase in engagement in this group may have been part of psychological strategies to manage the stress of lockdowns. This finding is significant given that females had a higher risk of experiencing poorer mental health during COVID-19 [8,19].

However, other groups showed initial changes during the first lockdown with these changes being attenuated relative to their counterparts in the second and third lockdowns. Younger adults were more likely to increase their engagement during the first lockdown, with evidence from studies showing their engagement was higher than older adults in the similar stage of the pandemic [6,12,13]. But the age difference in engagement changes was not seen in later lockdowns, suggesting that the momentum of changes was perhaps more noticeable in the first lockdown. Similarly, people with a mental health condition showed changing arts behaviours (both increases and decreases) during the first lockdown as reported elsewhere [6], but no association was found in later lockdowns. Further, people with a physical health condition were more likely to have reduced their engagement during the first lockdown, possibly due to higher stress and incidence of experiencing serious symptoms of the COVID-19 virus. Also more likely to have reduced engagement were those who were not employed, which could be due to more time spent on job searching especially when job offers became limited and financial worries grew as the pandemic developed. These findings echo other studies during the COVID-19 pandemic [6,11], yet followed similar changing patterns to their counterparts in later lockdowns. This suggests that the disruption of the first lockdown had the most noticeable effect on behaviours for these groups, but that their trajectories then aligned with their peers’ in subsequent lockdowns, supporting research showing greater psychological and behavioural reactivity to events amongst these groups at the start of the pandemic [20,21].

When exploring these changes for subsequent lockdowns in greater detail, further nuance about people’s arts behaviours is revealed. As shown in the Figures and the figures from S1 File, 2 in 5 people who had increased their engagement during the first lockdown maintained or further increased their engagement in subsequent lockdowns. For these people, it is likely that the first lockdown had acted as a strong catalyst for changes in typical arts engagement behaviours. The maintenance of increases could be explained by factors such as increased availability of online opportunities, modelling of behaviours from others experiencing lockdown both within communities and from celebrity figures, greater provision and marketing of free home-based activities (including online tutorials and resource packs), and increased awareness of the benefits of arts engagement to support mental health during the pandemic. The continuation of some of these events and opportunities in subsequent lockdowns may have continued to support the changes that people made in first lockdown, with the similar circumstances of those additional lockdowns recreating the environments that had first prompted the change. Alternatively, behavioural changes made during the first lockdown might have become persistent. A robust study has shown that new behaviours could become a habit on average after 66 days [22]. This suggests that the 11-week first lockdown might have been long enough for new arts behaviours to become routine. In contrast, 93% of people who had decreased their engagement during the first lockdown and maintained this lower engagement or further decreased in subsequent lockdowns. For them, new barriers might have arisen such as the lack of in-person community arts opportunities and lack of goals such as performances and exhibitions to work towards. In addition, while increases in online artforms emerged during the pandemic had made the arts more accessible, digital illiteracy and unequal access to reliable internet might have created additional barriers for some demographic groups. These changes have been shown to have affected factors relating to individual capabilities, opportunities and motivations, all of which have previously been shown to associate with individual characteristics such as mental health and age [23–26].

For a small proportion of people though, there was no marked change in their engagement levels during first lockdown, but there was during subsequent lockdowns (4% of individuals were “about the same” in first lockdown and then increased engagement in second lockdown, whereas 72% were “about the same” in first lockdown and then decreased engagement in second lockdown, with similar figures for third lockdown). These groups were likely affected by factors relating to changes in societal context and environment, such as different restrictions implemented across different lockdowns (e.g. children were allowed to go back to school in the second and third lockdowns). Given that it was more common for people to decrease rather than increase their engagement for future lockdowns, these changes might have encouraged engagement in other activities which displaced leisure time available for arts engagement. Further, the repeated lockdowns might have brought a sense of “pandemic burnout” where people felt physical and emotional exhaustion, and hence led to collective behavioural shifts. Some groups were more likely to exhibit this pattern of behaviours, including people who were from ethnic minority backgrounds, people with lower educational attainment, and those with higher household income. For people of ethnic minorities, they were more likely to decrease their engagement in the third lockdown relative to the first lockdown. One possible explanation could be related to the economic impact of the COVID-19 pandemic on ethnic monitories. It has been shown that ethnic minorities in the UK had a higher probability to experience job loss during the COVID-19 lockdown and were less likely to enjoy employment protection including furloughing [27]. As a result, the three-month lockdown in the third lockdown might have manifested such employment/financial struggle amongst ethnic minorities, and encouraged them to use their spare time to seek jobs to maintain their living and hence reduce their time engaging in leisure activities including arts and creative activities. With regards to education, in line with previous analyses which suggested that people with higher education tended to have a greater engagement rate compared to those with lower education [6,12,13], our findings showed that they were also less likely to reduce their engagement in later lockdowns. However, the engagement levels by household income showed a different pattern. People with higher income were more likely to reduce their engagement in later lockdown (which was also reflected in a previous study [11]; most likely due to high work demand and less free time for leisure activities or enjoying other alternatives to spend their leisure time).

Finally, there were groups that were less likely to experience changes in arts engagement over the three lockdowns and more likely to show a stable pattern of arts behaviours. For instance, older adults aged 60 or above were more likely to maintain the same level of engagement than to increase their engagement during first lockdown, suggesting a stable pattern of arts behaviours compared to working ages adults. Previous research supported this and suggested that people in this age group might be more likely to stick to their routines, and that the lockdown did not affect their day-to-day lives as much as for other age groups and thus did not alter their behaviours as much [28]. Also, this group did not experience the same levels of worsening mental health, so it is possible that this group did not draw on the arts so prominently to support their coping and emotion regulation [19,28–30]. Furthermore, adults living with children were more likely to maintain their engagement levels during lockdowns, suggesting their ongoing efforts in engaging in the arts with their children or using the arts as a positive coping strategy to manage their emotions [31,32]. Results on the pattern of engagement levels by living areas were inconclusive, further analysis is required to indicate the relative change.

This study has a number of strengths. The analysis was based on a large and heterogenous sample across major socio-demographic groups and the analyses were weighted to population proportions. However, this study was not without limitations. First, while our data was weighted to proportion of age, gender, ethnicity and education obtained from the ONS [15], it was not a random sample, so we could not rule out the possibility of biases due to other characteristics related to survey response not being accounted for in the weighting procedure. Further, the analysis relied on self-perceived changes in arts engagement, so participants might have experienced recall bias. Future research using a more objective measure (such as time use in the engagement) is encouraged. Also, the main focus of our study was home-based arts engagement in lockdowns; the patterns and predictors of outdoor arts engagement or engagement outside the lockdown periods are likely to be different. As we only focused on engagement during lockdowns, future research is needed to investigate how patterns of engagement might have changed during periods when lockdown restrictions were eased. Finally, although we used a rich measure of arts engagement in our survey, it is possible that more diverse activities were not included in our definition, potentially meaning that we did not fully capture all creative engagement of participants.

## Conclusions

Overall, this study examined changes in people’s arts engagement across three national lockdowns in England and identified the predictors of changes in arts engagement levels. Echoing previous analyses, we found that certain groups such as young adults were more likely to increase their arts engagement during first lockdown, other groups including people with a physical health condition and those who were not employed were more likely to decrease their engagement, and other groups such as females, people with a mental health condition, and people with higher educational qualifications showed changes in both directions.

However, our findings extended this by showing that some of these observed changes seemed to have been carried over in the second and third lockdowns for groups like women and people with higher education levels, whereas others such as young people, people who were not employed and those with a mental or physical health condition showed the same pattern of change in engagement as their counterparts in later lockdowns. Overall, two-fifths of people who increased their engagement during first lockdown maintained this increase in subsequent lockdowns, and 93% of those who had decreased their engagement continued with this lower pattern of engagement. For these groups, the pandemic may have affected barriers and motivations to engage and set new long-term cultural behaviours. The results have important implications for the future work of cultural organisations focused on delivering home-based arts activities whether through online provision or the sale of resources such as crafts. But they also have implications for the broader cultural sector as they may indicate new profiles of audiences for specific arts activities who need more targeted activities over the coming years.

Our findings also show groups that did not show much change in behaviours in first lockdown but did in a subsequent lockdown, including people with lower educational qualifications and ethnic minorities. This suggests the importance of continued monitoring of the experiences of these groups as the pandemic continues as there is the potential that this audience demographic could contract further as the pandemic continues and revert to the pre-pandemic levels. Specifically, more research to understand how changes in barriers to participation have shifted will be important. This work will need to consider not just individual motivations but also the wider societal context created by the pandemic including changes to cultural organisations, cultural funding and the creative workforce.

## Ethics

The study was approved by the UCL Research Ethics Committee [12467/005] and all participants gave written informed consent.

## Supporting information

S1 File (DOCX)
