# Supplementary figures and images for: Comparisons of home-based arts engagement across three national lockdowns during the COVID-19 pandemic in England
Source: PLoS One. 2022 Aug 31;17(8):e0273829. doi: 10.1371/journal.pone.0273829 (PMC9432750; doi:10.1371/journal.pone.0273829)

# **Supporting Information**


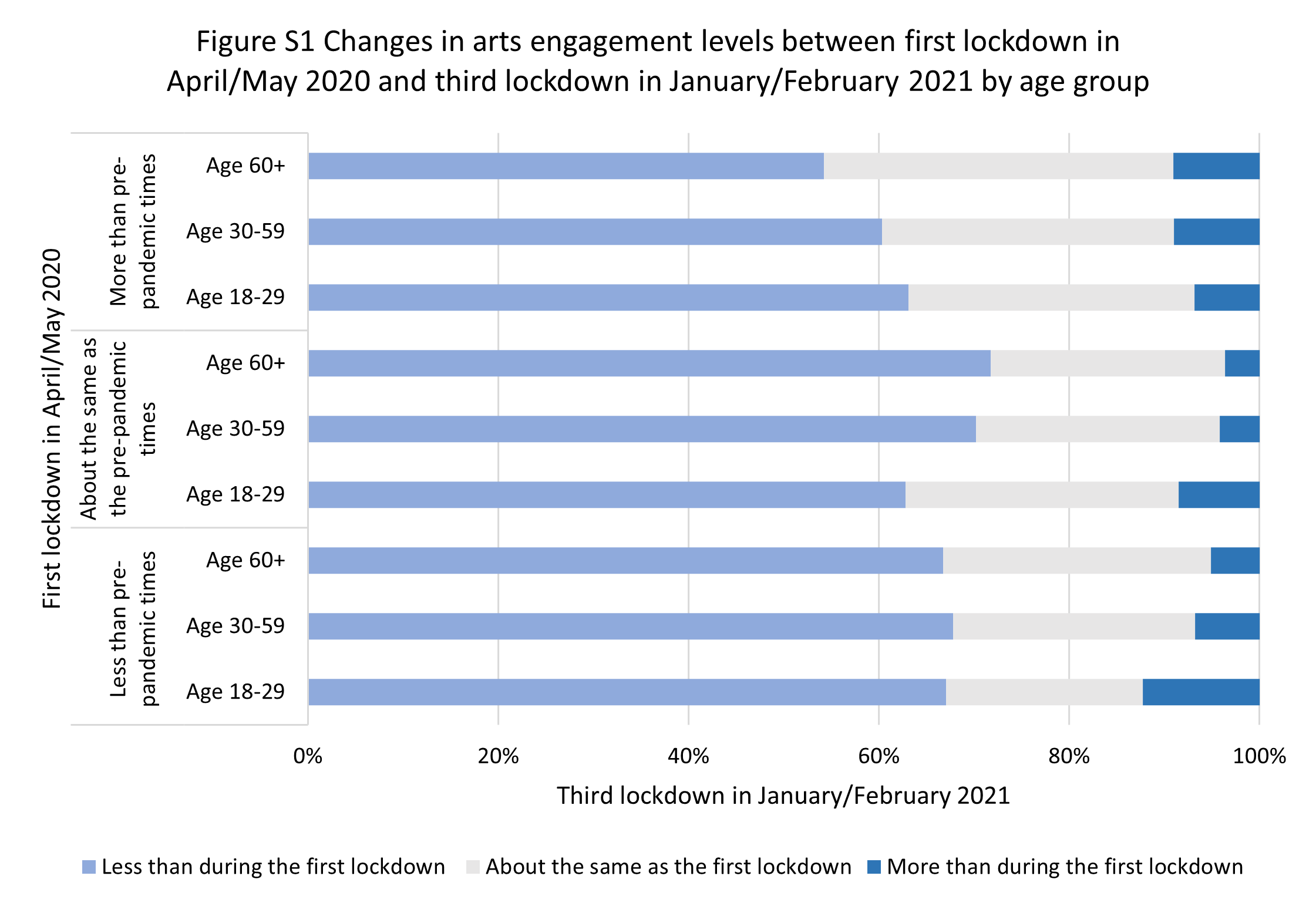


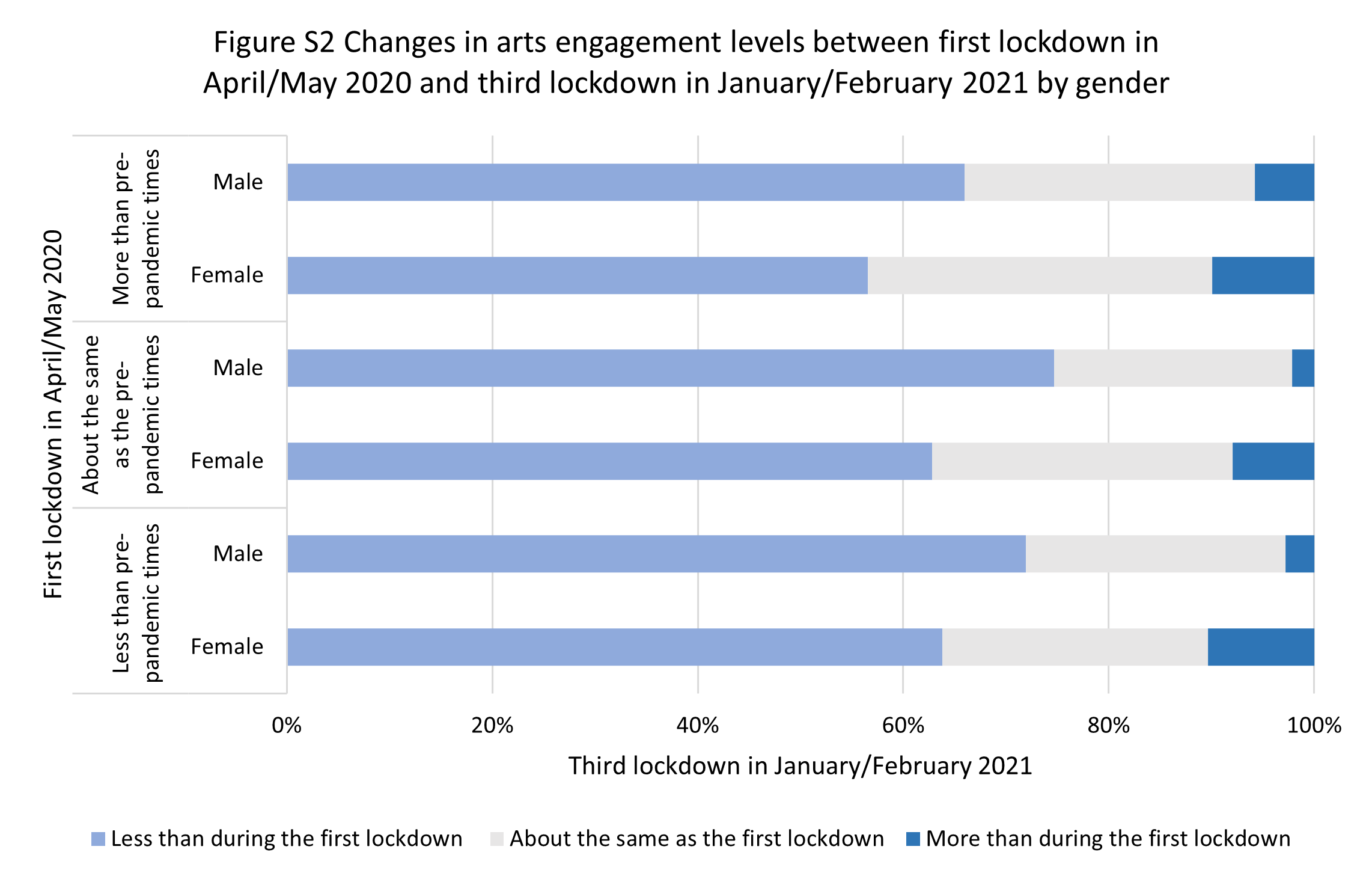


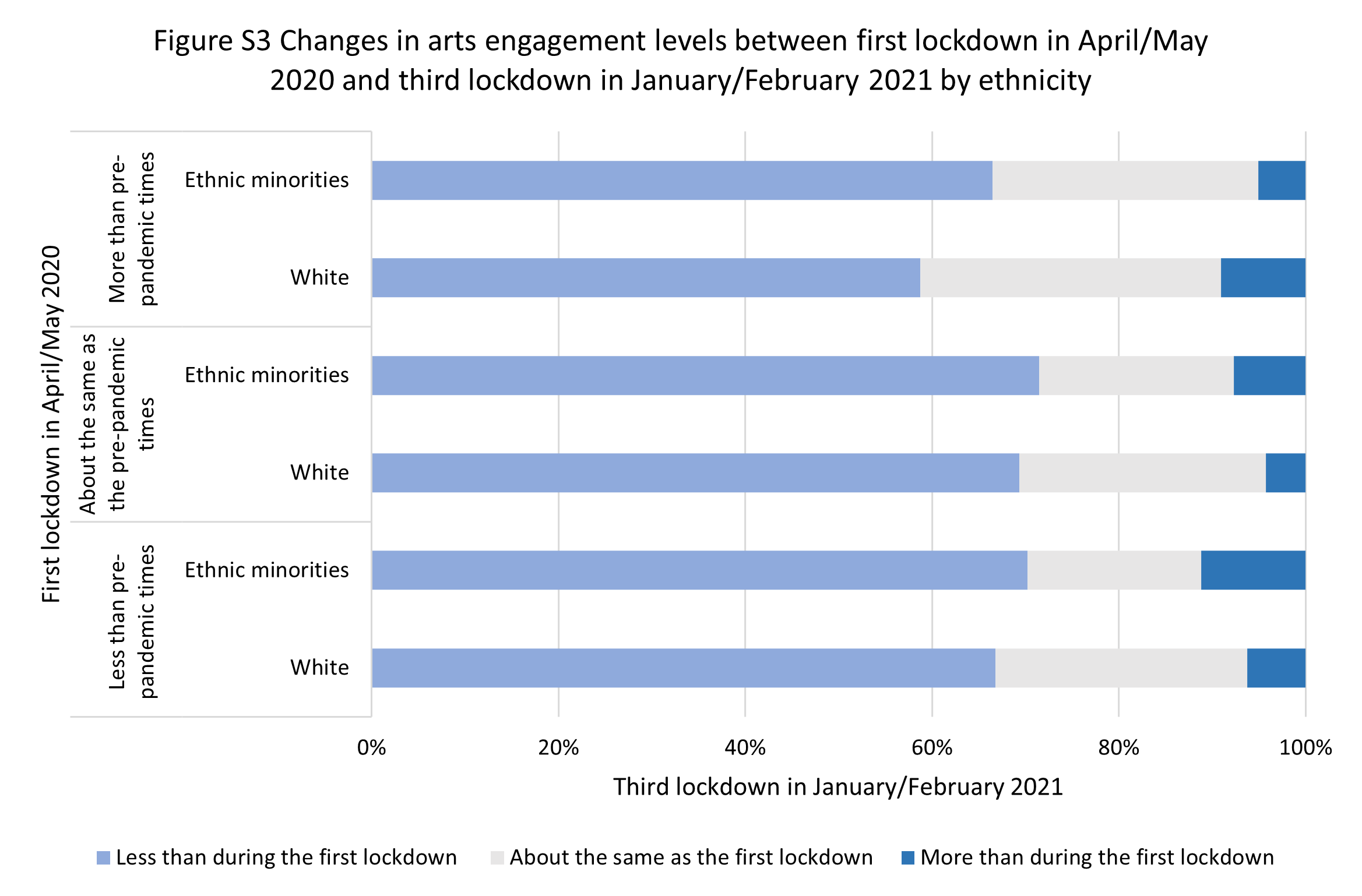


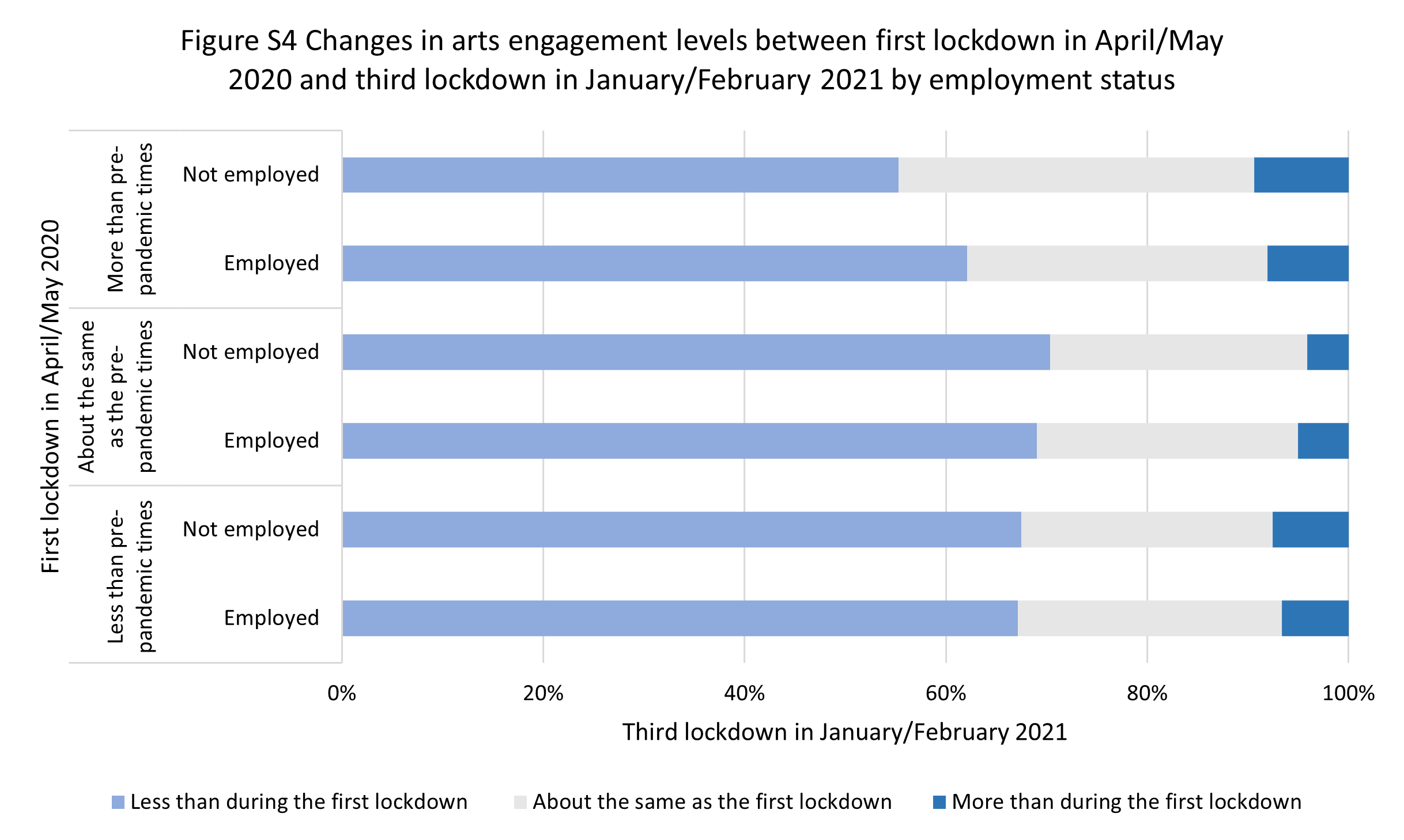


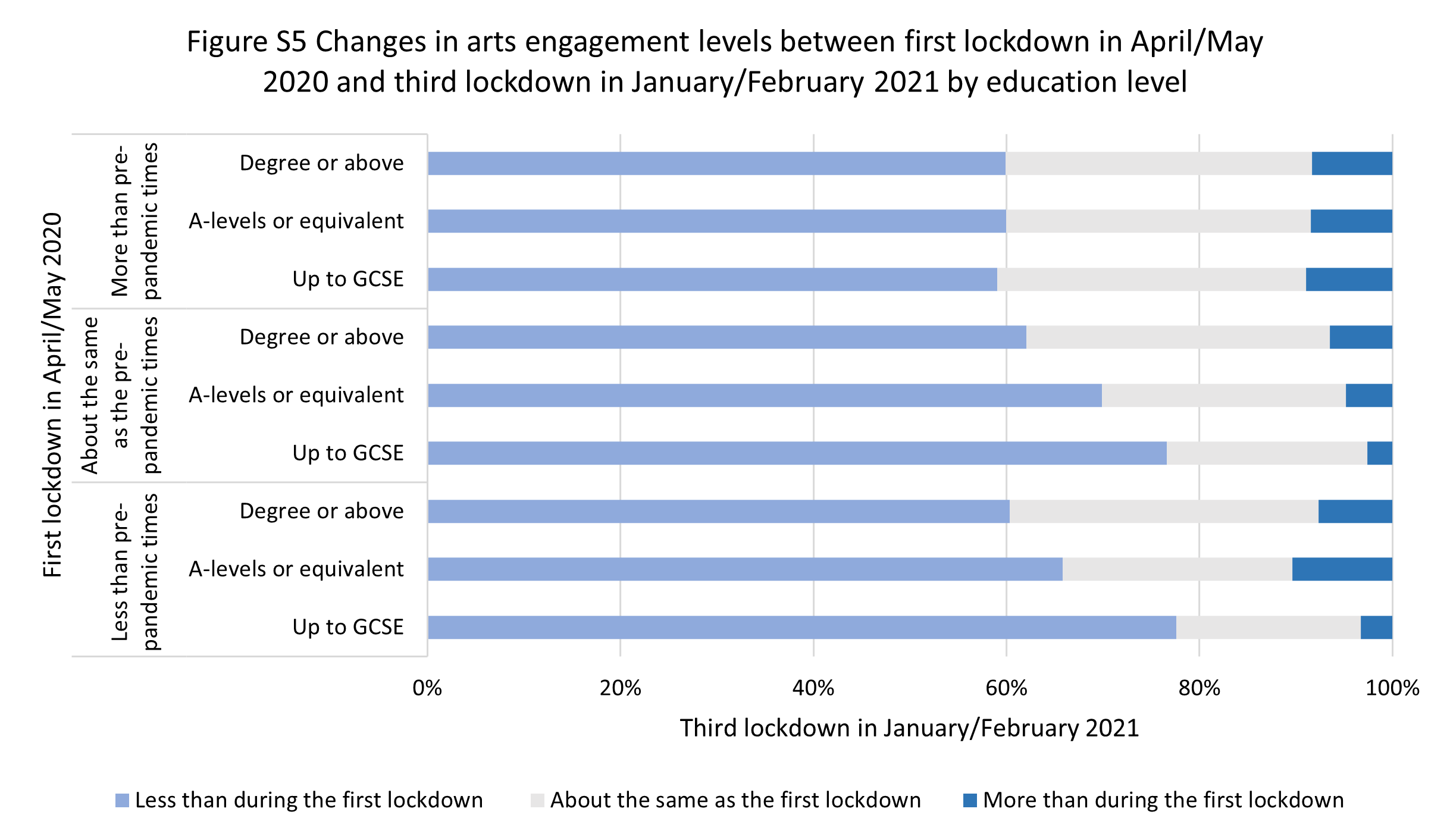


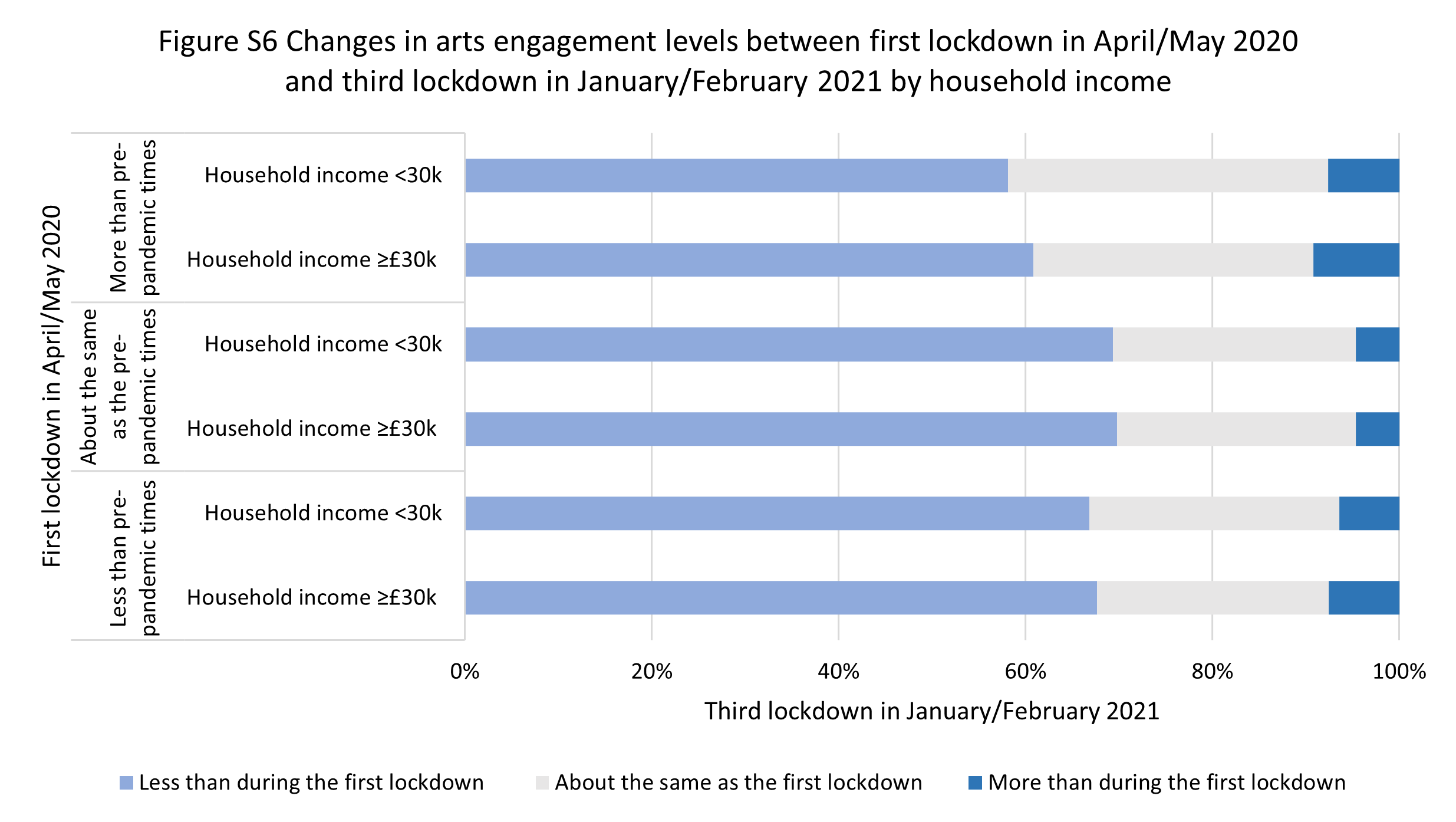


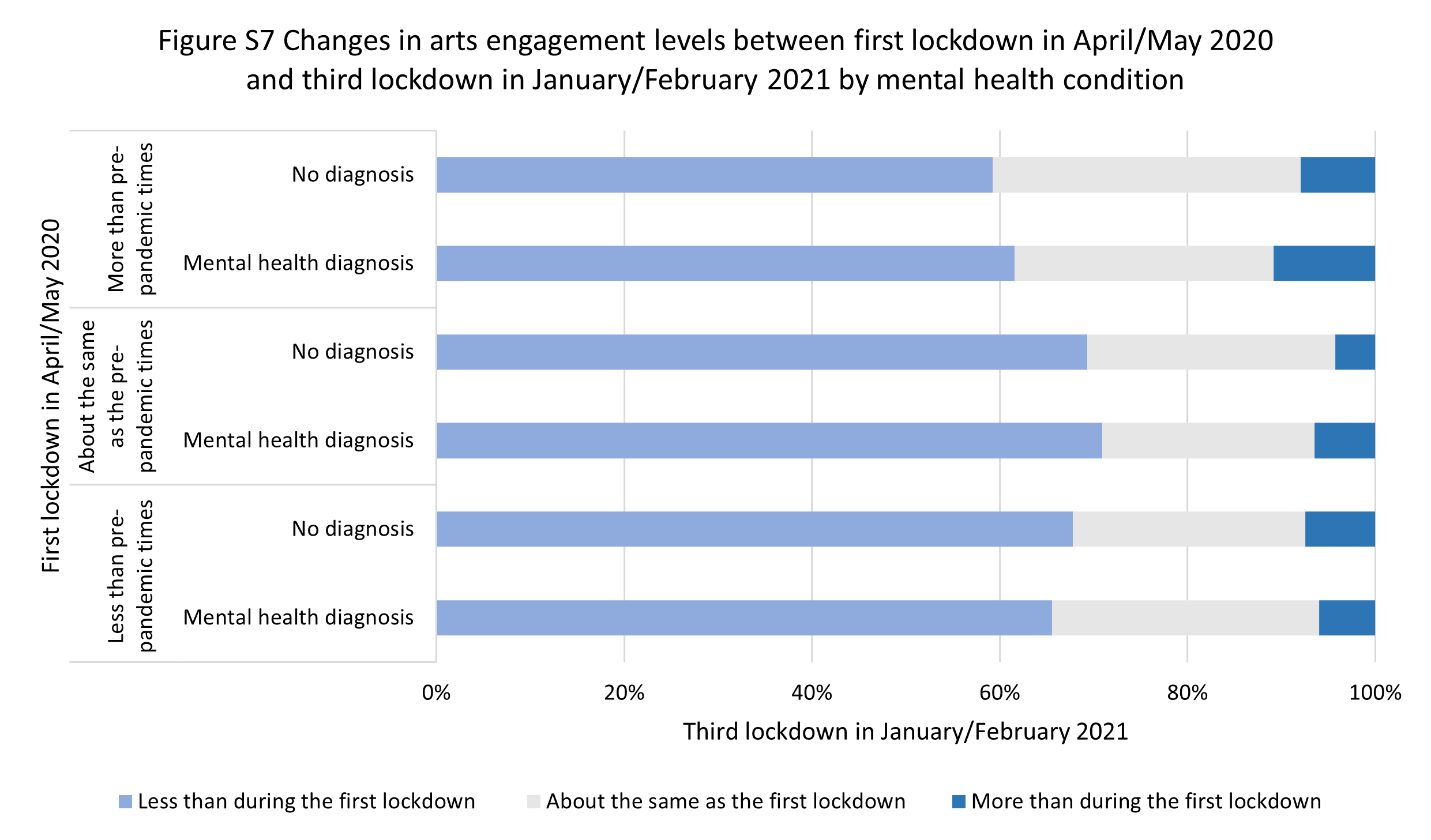


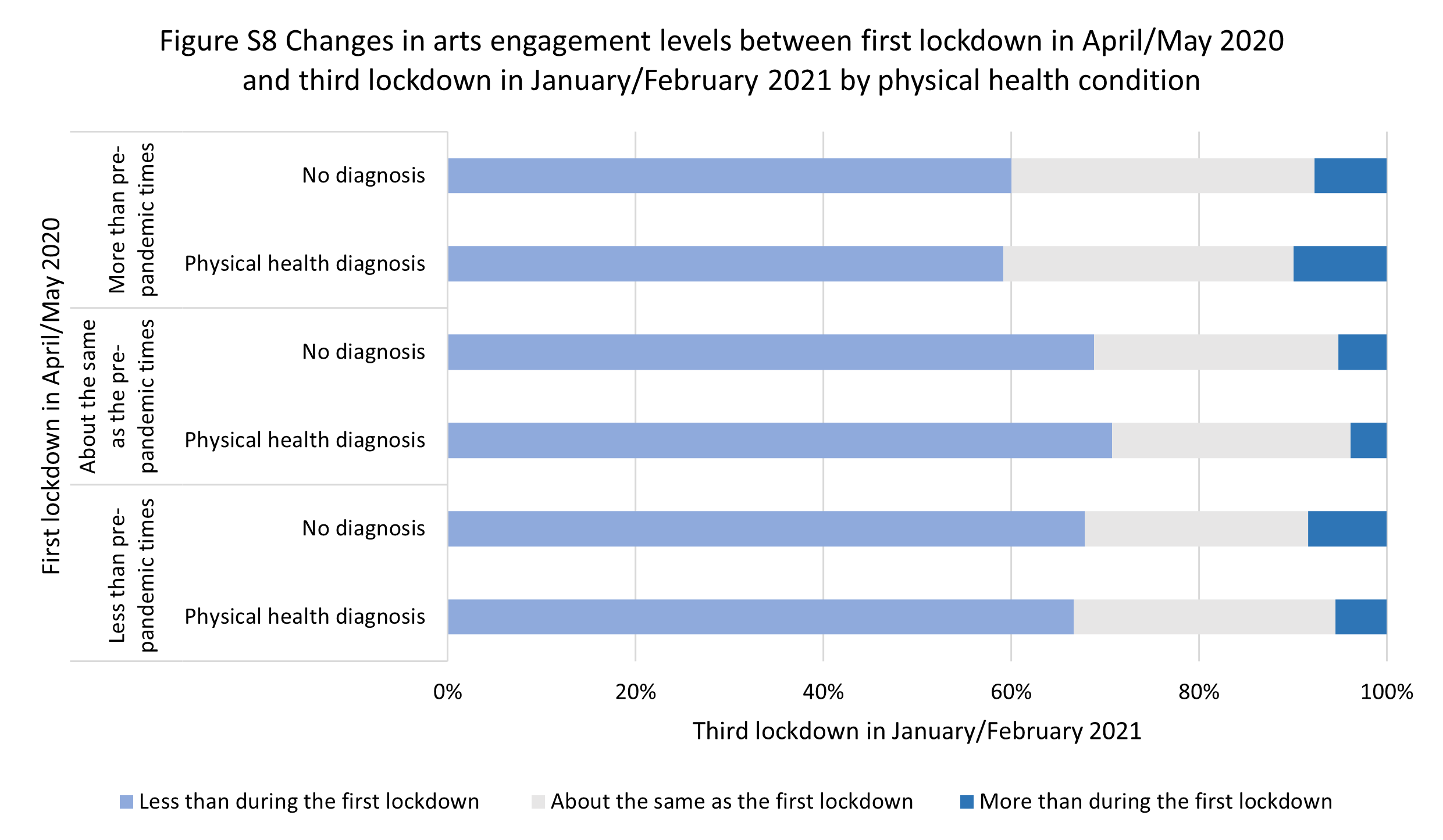

Supplement: S1 File — (DOCX) [file pone.0273829.s001.docx]
